# The experience of disagreement between students and supervisors in PhD education: a qualitative study

**DOI:** 10.1186/1472-6920-13-134

**Published:** 2013-09-28

**Authors:** Ronny Gunnarsson, Grethe Jonasson, Annika Billhult

**Affiliations:** 1 Research and development unit of the county Södra Älvsborg, Sven Eriksonsplatsen 4, Borås 503 38, Sweden; 2 Cairns Clinical School, School of Medicine and Dentistry, James Cook University, Cairns, Australia; 3 Department of public health and community medicine, Institute of Medicine, The Sahlgrenska Academy, University of Gothenburg, Gothenburg, Sweden

**Keywords:** PhD education, PhD student, Supervisor, Research, Disagreement, Higher degree education

## Abstract

**Background:**

PhD supervision is mostly individual and disagreement between supervisors and PhD students is a seldom-discussed topic at universities. The present study aimed to describe the experience of disagreement between PhD students and supervisors.

**Methods:**

Nine supervisors and seven PhD students from Sweden and England were interviewed using a video recorder. The recorded material was analysed using inductive content analysis.

**Results:**

Disagreements in PhD education can be described with the overarching theme: the nature of the disagreements changes over time. Five categories emerged to describe the variations of the experiences: involvement in important decisions, supervisors not being up-to-date, dubious advice from supervisors, mediating between supervisors, and interpersonal relationships.

**Conclusions:**

There is a gradual shift in competence where PhD students may excel supervisors in subject knowledge. Early disagreements may indicate immaturity of the student while disagreements later may indicate that the student is maturing making their own decisions. Consequently, disagreements may need to be addressed differently depending on when they occur. Addressing them inappropriately might slow the progressions and result in higher attrition rate among PhD students. The five categories may be elements in future PhD supervisor training programs and should be further evaluated for their importance and impact on PhD education.

## Background

Supervision in higher education is a pedagogical challenge [1]. It is an old phenomenon as doctoral programs were formed over 100 years ago, starting in Germany and then spread to other countries and universities. Johns Hopkins and Clark Universities were among the first to issue PhD diplomas [2,3]. PhD education does not, however, take place without some disagreements between supervisors and students.

Earlier research points out areas for improvement in PhD education. PhD education e.g., in the Netherlands, has faced criticism regarding the long process towards completion, a high percentage of non-completion, and inadequate funding. Supervision problems exist such as an inadequately low meeting frequency and depth, resulting in a stressful and lonesome PhD-education as well as frustrated supervisors [4]. Critique of PhD education has been documented by the Evaluation Committee on PhD education in Sweden [5]. A disproportionately lengthy time from registration to graduation, a high mean age of doctoral students, and a high attrition rate from PhD education by registered students, were issues brought up as areas of dissatisfaction. Discontent with the supervisor was documented by twenty-five per cent of the students, and as many as one in ten seriously considered a change of supervisor [6]. Furthermore, attrition from PhD education has been measured, and concluded to be costly and resource demanding [7].

Supervision in higher education appears to be a lonely task, as it is mostly an activity involving only the supervisor and the student. Supervisors may discuss supervision with colleagues, but these discussions are more of a formal nature seldom elaborating on individual cases in the task of supervising PhD students [8]. Therefore, it is of interest to investigate and document PhD student and supervisor experiences that may perhaps lead to decreased attrition levels and more satisfied students and supervisors within PhD education.

Since no one, to our knowledge, has specifically studied the experience of disagreement between supervisors and students, the aim of the present study was to describe the experience of disagreement between supervisors and PhD students in the context of higher education at university.

## Methods

### Sample and context

A strategic sample to achieve variation in age and experience in PhD education was chosen to include nine supervisors: one woman and eight men. Five supervisors were tenured members of the staff at a British university interviewed in England, and four were employed at a Swedish university and interviewed in Sweden. Their experience of supervising varied from 2 to 30 students supervised in PhD programmes. Seven PhD students were included, four women and three men. Five were enrolled at a British university, and thus interviewed in Britain. Four of these students were interviewed as a group. Two were enrolled at a Swedish university and interviewed in Sweden. The students had between 1–5 years experience in the PhD programme. The study was conducted in the context of higher education at university.

Research ethics is regulated in law in Sweden. Studies with no intention to harm or influence humans, only being interviews or questionnaires, not dealing with sensitive personal information such as sexual orientation, political views or similar sensitive information do not need to be evaluated by a formal ethical review board. Thus, this study was not submitted to a formal ethical review board. However, the authors made efforts to ensure the study was performed in accordance with the guidelines of the Declaration of Helsinki as revised in 2000. The informants in this study all had own experience in research and could be expected to understand the given information. The informants were told that they could withdraw participation at any time without stating any reason. Furthermore, each informant signed a written informed consent before the interview began.

### Data collection

Data were collected by means of a digital video recorder. The first researcher, RG, briefly introduced himself and then gave a short presentation of the practicalities of video recording, for example avoiding looking straight into the camera. The camera was placed on a tripod and switched on. RG afforded the informant a short period of time to get used to the camera. The interview started with the opening phrase: “Please tell me of a situation where you and the PhD student/supervisor (depending on the informant being interviewed) had different views on an issue”. The informant was then encouraged to elaborate on the phenomenon of disagreement between the supervisor and PhD student. Interviews lasted as long as the informant added substance pertaining to the research question, roughly 35–60 minutes.

### Data analysis

When human experience of a phenomenon is to be described, a qualitative research method is suitable [9]. Content analysis, a qualitative research method used to quantify phenomena systematically [10], was used to analyse the present material. The main aim of the analysis was to condense the extensive material into a few content categories by way of inductive content analysis as described by Lauri & Kyngäs [11]. Analysis was carried out in three phases: preparation, organization and reporting. In the preparation phase, the raw video material was watched repeatedly until a sense of the whole was obtained. The organizing phase consisted of coding the data into main aspects of the video recordings, which were noted along with the location on the timeline in the raw video material. These were then transferred to a coding sheet and condensed categories were formed. Categories were then classified to fewer, higher order groups and finally, as last part of phase two, a general description, or overarching theme was formulated through abstraction. In phase three of the analysis, the analysis process and results of the study were reported [9].

## Results

The experience of disagreement between supervisors and PhD students in the context of higher education at university can be described with the overarching theme “the nature of disagreements changes over time”. Five categories emerged to describe the phenomenon of disagreement: important decisions, supervisors not being up-to-date, dubious advice, mediating between supervisors, and interpersonal relationships.

### Disagreements changes over time

Disagreement between PhD students and supervisors often exists during PhD education. PhD students experienced disagreements more often than did supervisors. Supervisors acknowledged that transient disagreements, but not conflicts, existed. Disagreement between student and supervisor could be aggravating and arouse strong emotions. However, as PhD education progressed, the nature of disagreements changed as the relationship between supervisor and PhD student developed. Sometimes supervisors found themselves being “outmanoeuvred and outthought” by the students as there was a shift in subject knowledge over the course of the PhD education. PhD students also matured, gained confidence and became more involved in important decisions regarding the direction of their project. They also acquired the possibility to identify dubious advice as time went on. Similarly, they learned to mediate between supervisors in an efficient way in order for the research to progress. Since the PhD education lasted several years, the interpersonal relationship between the supervisor and the PhD student developed, and they learned to balance critique and communication.

### Involvement in important decisions

Participation in key decisions was equally important to both supervisor and student. Important decisions could include changing the aims of the thesis, or choosing which analysis to use. The rationale for wanting to participate in important decisions differed for the PhD student and supervisor. Supervisors found that student influence on important decisions sometimes created problems:

“There have been occasions when I have found students making major decisions about the project without discussion”.

Another supervisor expressed worry because the student wanted to perform an analysis unknown to the supervisor:

“*He* [the student] *made a very strong case that this was the methodology to use. In this case, because I didn’t understand the methodology, I wouldn’t have been able to detect that because of my lack of knowledge… … so I felt uncertain”.*

In this case, the supervisor did not mind that the student wanted to make a major decision, but was rather concerned by the consequences, for the student, supervisor and department, if the analysis was flawed in its methodology. Furthermore, supervisors were concerned whether students were capable of carrying out the analysis. PhD students felt they matured as education progressed. At first they needed more support, but eventually felt confident enough to influence major decisions. One doctoral student expressed:

“…initially to do with what exactly would be in my PhD. Perhaps my feeling that the content of my PhD was being led by their research interests rather than what I wanted to do… …I ended up doing what I wanted to do. So it was fine in the end”.

### Supervisors not up-to-date

The supervisor was not always up to date concerning the project. PhD students felt they had the best knowledge of what was going on in the project. One PhD student expressed frustration, as his supervisors were not up to date:

“Okay, I have reached this result. And they’ll have kind of forgotten about what I had been doing up to that point. I know a little bit more especially about my own work than they do. So they’ll disagree on something that I had already dealt with”.

Sometimes the supervisors were not updated as they were discussing and disagreeing on matters that the PhD student had already dealt with and solved on his own as he gained confidence. The PhD student went on to say that the supervisors thought that he [the student] was being dismissive toward their objections, which was not the case. The supervisor, on the other hand, explained the difficulties of being a supervisor:

*“The student thinks about nothing else, and you* [the supervisor] *think about it sometimes for one hour every three or four weeks. So it is quite difficult in a PhD supervision session to get up to date, to get your mind up to speed as quickly as you need, so you occasionally find yourself being outmanoeuvred, and outthought by the student”.*

### Dubious advice

PhD students claimed that supervisors sometimes gave dubious advice. At times, the supervisor gave advice that was not well thought out. That led to lost time for the PhD student, trying to follow the path set out by the supervisor. One PhD student told of moments at the start of the PhD education where the supervisor said:

*“-Yeah try that, why not. And he* [the supervisor] *just thought about it at just that instant. Especially when I started I thought, well, he is a professor… … he is experienced, he knows about these things, so I kind of treated every suggestion he made as a very serious thing that I should definitely pursue until I had exhausted that possibility”.*

However, as time went on, the student came to ignore some of the supervisors’ suggestions as he realised that the supervisor had not reflected on the idea thoroughly. The supervisors, on the other hand were not always confident of which ways were correct:

“*Sometimes I have been quite sure that my way was right. But that is very rare. It occurs more often that you try to find something that is common, and that what is right or wrong is very difficult to say”.*

Another supervisor expressed how it was not possible to know everything. Keeping an open mind as a supervisor allows for new knowledge. One way of dealing with uncertainty could be to ask someone else:

“Methodological questions can be resolved in another way. Concerning different methods, or adding methods, we often ask a third person… …it is good to get the view of a third person”.

### Mediating between supervisors

The student may have to mediate between supervisors. As the PhD student had two, sometimes three supervisors, they often had different views making different comments and giving different advice. The student then had to act as mediator to unify the members of the team. If not, research would stagnate. A PhD student noted this regarding the writing of an article:

“In the case of writing an article, more than one supervisor is involved in the article. They are then co-authors and I am supposed to maintain a dialogue with everyone… … I then have to balance this”.

The student immediately took on the role of mediator, realising that it was in his best interest to keep supervisors informed in order to get the article published. The student had to learn of efficient ways to mediate between supervisors. Another PhD student experienced difficulties in the collaboration between supervisors as they were at separate locations:

“I have an external supervisor at a different university … … I find it difficult to manage the relationship between my external supervisor and my internal supervisor here …”.

When commenting on this to the principal supervisor, she was told that managing different personalities was part of the education, and thus a learning experience.

Supervisors were also aware of the fact that it was always the student that had to mediate when supervisors held different views:

“*The two supervisors have completely different views, and the PhD student has to be the mediator. That can be very hard on the PhD student”.*

### Interpersonal relationship

Personal chemistry and emotions played a role in PhD education. Preconceived notions and stereotypic labelling of reactions on behalf of the student and supervisor existed from the student’s point of view:

*“There are emotions on both sides, and you don’t want to acknowledge that might happen. That actually you people* [supervisors] *might get emotional about it. I find it particularly difficult to believe that I would ever get angry with my supervisors for some reason”.*

This student expressed a notion that PhD work is mainly rational and based upon logic. At the same time, he knew that emotions existed on both sides.

Supervisors were aware that different personalities must be met differently. One supervisor reasoned that some PhD students might experience a challenge when opposed, while others just fell apart:

“*I don’t think that you should go along too much. The extent depends on the personality of the PhD student”.*

The supervisor expressed it as a balancing act to oppose and challenge the student just enough to get a proactive response rather than a collapse on the behalf of the student. Another supervisor spoke of the relationship with the PhD student as becoming a long lasting friendship even after the PhD education ended.

## Discussion

The findings of the present study imply that disagreement between PhD supervisors and students occurs and the nature of disagreements changes over time within the PhD education. When occurring, it can be condensed into five categories: important decisions, supervisors not being up-to-date, dubious advice, mediating between supervisors, and interpersonal relationships. Parts of this material can, with the informants’ written consent, be seen on the Internet [12].

### Discussion about results

#### The nature of disagreements changes over time

As PhD education continues several years, it is not surprising that the nature of the disagreements change over time. In what particular ways it changes over time was not expressed by the informants but our implicit impression was that it was mainly due to the student’s increasing knowledge and ability to make own decisions. Early disagreements may indicate immaturity of the student while disagreements later may indicate that the student is maturing making their own decisions. Consequently, disagreements may need to be addressed differently depending on when they occur.

The severity of disagreements, whether the disagreements were resolved or what impact they may have had on the research project, were not addressed in this study. It is possible that inclusion of supervisors/PhD students with experiences of serious disagreements may have yielded quite different results. Our view is that the disagreements in the present study were mainly not of serious character.

### Involvement in important decisions

Supervisors in the present study did not mind that students were involved in important decisions, but rather concerned for the consequences of such decisions. Since supervision is part of the supervisor’s career as well as the student’s, outcome is very important for the supervisor, and thereby, the department. However, Cullen et al. [13] noted that some supervisors left it up to the student to make major decisions. The students then, in retrospect, felt a general lack of moral support from the supervisor [13]. The PhD students in the present study claimed autonomy and a wish to influence major decisions, but not until having matured sufficiently to feel confident enough to do so, implying that supervising seems to be a balancing act as to the degree of influence by the supervisor.

The model adopted at the university also influences degree of student involvement in important decisions. Massachusetts Institute of Technology (MIT) has developed a model for contract research with the industry. In this model, the PhD projects are short term, limited in scope, and with clearly identified milestones for delivery [14] leaving the student with few possibilities to be involved in important decisions. Work is directed by the supervisor and the industrial sponsor, and the student is often employed by the sponsor after finishing the PhD-education. This model often provides financial security. Another model is adopted in Cambridge. It is up to the student to form his or her own project with guidance from the supervisor. To be able to influence the research project offers a powerful motivating factor for the student [14]. The freedom to choose research direction has also been highlighted in the model of the Vienna University of Technology, together with the requirements to have a PhD by publication, to let students work in shared offices towards joint deadlines, and to involve students in reviewing articles [15]. The supervisors are supporting and helping, but most criticism is given by external sources [15]. The freedom to define own research topic entails that the supervisor educate the student to take high-level decisions and become owner of the project [15]. The shared offices may help to socialize the students. Theories of socialization have been connected to the issue of attrition in doctoral education where inappropriate socialization may be related to students departing the PhD program [16].

### Supervisors not up-to-date

As supervisors often have more than one PhD student in progress, they cannot keep up to date the way students do. This creates disagreements as the student feels time is lost dwelling on issues already dealt with. As supervisor hours are scarce, this affects not only the student but the supervisor as well. Lauvås & Handal gives advice for optimal use of feedback in research supervision: “Do not try to conceal inadequate preparation” [17]. PhD students need to be able to thoroughly rely on advice and comments from their supervisor. Unacceptable work ethics such as trying to conceal inadequate preparation or adopting an attitude of neglect is not usually accepted by students [17].

### Dubious advice

A source of disagreement between supervisors and PhD students arises when the student receives dubious advice. Inadequate knowledge and skills of the supervisor is a known criticism of PhD supervision [18].

Students, in the present study, expressed concerns that dubious advice was time wasting. However, students must also mature in their abilities to judge what is correct and what is dubious. A physicist interviewed by Gumport [19] articulated the following:

“I try to teach them a set of skills. The biggest one is to know when you’re right and when you’re wrong. It’s common for them to miss it when they’re wrong. After a while they can see it. It’s intuitive partially” [19].

Although the student is ultimately responsible for his or her own work [20], and may feel the supervisor should have all the right answers, a more nuanced picture of dubious advice is realizing that there is in fact no manual and both the supervisor and PhD student learn during the process.

Supervisors in the present study adopted a humble attitude saying they were not always sure of what was right or wrong. This is in accordance with Delamont et al. [21] who discussed the nature of academic supervision. They mean that the skills of academic judgement, evaluating research and assessing publications, must be learned over the course of a career and there is no manual with instructions. Confidence is of fundamental importance in the supervisory process [21]. The supervisor needs to be confident in the student and the student needs to feel confident in the overall judgment of the supervisor, but the confidence has to be ‘informed’ not blind faith [21]. It is then only natural that a PhD supervisor is not always precise when providing advice, but sometimes gives dubious advice.

Halse [22] has investigated the question of learning generated through the practice of doctoral supervision. She described that supervisors, like those in our study, learn to become a supervisor by doing it and thus represent the participatory and practice-based learning theory [22]. In Sweden as well as in many universities across Europe, Australia and New Zeeland, professional development programs for doctoral supervisors have become mandatory. Even experienced professors should get a “driving licence” for PhD supervision. This formal training is based on the idea of the transmission model of learning. This model implies that good supervision is accomplished by attending a course, thus presuming that deficits in supervisor’s expertise can be remedied through formal, structured transmission of knowledge from instructor to learner/professor [23].

Previously, some supervisors tended to perceive the students as causing difficulties, making the assumption that the same supervisory resources and structures were adequate for all students; thus the structural and systemic problems that may exist were made invisible [24]. Now supervisors’ and universities’ role have been penetrated in many studies. The idea of a learning alliance has been proposed, meaning that the goal of doctoral supervision is praxis and involves a learning alliance between each student, supervisor and university grounded in mutual respect to ensure a high quality PhD education [25].

### Mediating between supervisors

As a rule, PhD students have one principal supervisor and one or more co-supervisors [20]. The persons involved in PhD studies can therefore be seen as a team, with different roles. The findings from the present study suggest that it is most often the student who must take on the role of mediator between supervisors when needed. One supervisor even thought that managing different personalities could be considered a part of PhD education. This view was, however, not shared by all supervisors, whereby one supervisor expressed that mediating could be hard on the student. In any case, students are ultimately responsible for their work, and thereby responsible for making progress [20]. By nature, the mobility of researchers is fairly high, creating geographically distributed teams and further difficulties. Disagreements in widespread teams are common [26], confirmed in the present study by students expressing difficulties in managing cooperation and mediating their in-house supervisor with other supervisors located at another university.

### Interpersonal relationship

Both supervisors and students in the present study acknowledge that their relationships affect the process of the PhD education. Different personalities seem to require different behaviour. The relationship between the supervisor and student changes as the PhD education progresses. Handal & Lauvås [1] noted that the level of competence may shift from the supervisor to the PhD student, as the student in time acquires knowledge superior to that of the supervisor in his/her narrow field. For some supervisors, this is an affirmative event, but for some it may be threatening.

Disruptions in the relationship between student and supervisor can, according to Delamont et al. [21], be intellectual, personal or structural. In the case of intellectual or personal disruption, a change of supervisor is advisable as soon as possible. A structural disruption, such as the death of a supervisor, or more commonly, the transfer of a supervisor to another location, may be detrimental to the process in PhD education. Some of the supervisors in our study were careful to establish intellectual and personal boundaries between them and their students, whereas others did not mind to be personally involved with their students and eventually became friends. The duality in the supervising situation: to support and demand at the same time may generate tensions and strain within the tutoring relation [27]. Constructive criticism is necessary if good work is to be produced, since this assists students in thinking analytically and moving forward in their development. Supervisors’ educational development throughout the Western world is located largely within an administrative framework that emphasises supervisors’ and students’ mutual roles and responsibilities [28]. Yet some of these programs focus solely on the administrative roles and responsibilities of supervisors, attempting to provide technical “fixes” that deny the genuine difficulties and complexities involved in supervision relationships [28].

### Discussion of methods

Limitations of the methodology used in the present study include the notion of the researcher’s knowledge of the phenomenon and openness. Human science research builds upon intersubjectivity [29]. The researcher must acknowledge the fact that he or she interacts with the informant and thereby also has the possibility to influence the thoughts and expressions of the informant. It is by acknowledging and reflecting on past knowledge of the phenomenon that the researcher can keep an open mind, thus refraining from dominating the interview. It has been the aim of the researchers to adopt an open mind throughout data collection and analysis of the present study. Recognizing that RG, GJ and AB are supervisors in higher education, we reflected on our past knowledge and throughout the analysis striving to set it aside to benefit openness. If we have been successful, these limitations turn to strengths, as we thereby convey the experiences of the informants in a nuanced manner.

Another methodological concern is validity of the results [30]. It has been the aim of the researchers to include informants able to convey experiences relative to disagreement between supervisors and students of higher education at university. However, as their experiences are contextual and from individual perspectives, it has also been the aim of the researchers to form a broader understanding of the phenomena, bearing in mind that no truths are presented, merely the experiences of the informants, sensitive to the receiver of the message.

Furthermore, we strived for a high level of trustworthiness of the results in the present study by reflecting on the concepts of credibility, dependability and transferability [31]. Credibility was attained through carefulness in selection of the context, the informants and data collection. Furthermore, videotaping the interviews enabled preservation of silent expressions such as body language. Dependability was strived for by truthfulness when collecting the data in a predetermined, condensed time frame. Describing the context, informants, process of gathering data, and giving the methodological description and presentation of data as complete as possible facilitated transferability.

The researchers had an intention to achieve a strategic sample with variation in age, gender, and experience in PhD education. One female and eight male supervisors agreed to participate. It is possible that a more even spread in gender among the supervisors, would have influenced the results.

Four students were interviewed as a group due to practical circumstances. It is possible that interviewing them one by one may have changed the type of data received. However, the interviewer focused on an open attitude, leaving room for everyone to comment and freely speak about their experiences.

## Conclusions

Disagreements between supervisors and PhD students occur. Since PhD supervision seems to take place “behind closed doors” it is important to illuminate the experiences of both PhD students and their supervisors. This study shows that the nature of disagreement changes over the course of the PhD education. There is a shift in competence where PhD students excel supervisors in subject knowledge. Early disagreements may indicate immaturity of the student while disagreements later may indicate that the student is maturing making their own decisions. Consequently, disagreements may need to be addressed differently depending on when they occur. Addressing them inappropriately might slow the progressions and result in higher attrition rate among PhD students.

The five categories should be further evaluated for their importance and impact on PhD education. Raising the question of disagreements between PhD students and supervisors, in PhD supervision training, using the five categories, may lead to cost effectiveness by decreasing attrition rates and aid in reducing individual distress during PhD education.

## Competing interests

The authors declare that they have no competing interests.

## Authors’ contributions

RG planned the study, performed the interviews, and participated in the analysis of data and draft of the manuscript. GJ and AB participated in the analysis of data and draft of the manuscript. All authors read and approved the final version of the manuscript.

## Pre-publication history

The pre-publication history for this paper can be accessed here:

http://www.biomedcentral.com/1472-6920/13/134/prepub
